# 3D FEM Analysis of High-Frequency AlN-Based PMUT Arrays on Cavity SOI

**DOI:** 10.3390/s19204450

**Published:** 2019-10-14

**Authors:** Wenjuan Liu, Leming He, Xubo Wang, Jia Zhou, Weijiang Xu, Nikolay Smagin, Malika Toubal, Hao Yu, Yuandong Gu, Jinghui Xu, Denis Remiens, Junyan Ren

**Affiliations:** 1 State Key Laboratory of ASIC and System, School of Microelectronics, Fudan University, Shanghai 201203, China; 2 Université Polytechnique Hauts-de-France, CNRS, Université Lille, ISEN. Centrale Lille, UMR 8520–IEMN—Institut d’Électronique de Microélectronique et de Nanotechnologie, DOAE—Département d’Opto-Acousto- Électronique, F-59313 Valenciennes CEDEX 9, France; 3 School of Electrical and Electronic Engineering, Nanyang Technological University, Singapore 639798, Singapore; 4 Institute of Microelectronics, Agency for Science, Technology and Research (A*STAR), Singapore 138634, Singapore

**Keywords:** PMUT, 3D FEM, high frequency, AlN, array

## Abstract

This paper presents three-dimensional (3D) models of high-frequency piezoelectric micromachined ultrasonic transducers (PMUTs) based on the finite element method (FEM). These models are verified with fabricated aluminum nitride (AlN)-based PMUT arrays. The 3D numerical model consists of a sandwiched piezoelectric structure, a silicon passive layer, and a silicon substrate with a cavity. Two types of parameters are simulated with periodic boundary conditions: (1) the resonant frequencies and mode shapes of PMUT, and (2) the electrical impedance and acoustic field of PMUT loaded with air and water. The resonant frequencies and mode shapes of an electrically connected PMUT array are obtained with a laser Doppler vibrometer (LDV). The first resonant frequency difference between 3D FEM simulation and the measurement for a 16-MHz PMUT is reasonably within 6%, which is just one-third of that between the analytical method and the measurement. The electrical impedance of the PMUT array measured in air and water is consistent with the simulation results. The 3D model is suitable for predicting electrical and acoustic performance and, thus, optimizing the structure of high-frequency PMUTs. It also has good potential to analyze the transmission and reception performances of a PMUT array for future compact ultrasonic systems.

## 1. Introduction

Ultrasonic transducers have been widely used for medical imaging [1], nondestructive testing [2], rangefinders [3], gesture recognition [4], fingerprint systems [5], etc. High-frequency ultrasound transducers (>10 MHz) have attracted more attention for their applications in in vivo and high-resolution imaging [6,7], such as cardiovascular imaging [8], intraoperative catheters [9], and so on. Ultrasonic transducers based on piezoelectric ceramics [10] are bulky, lead-containing and low-frequency limited. They are not suitable for 2D arrays because of the poor consistency among the elements. Recently, micro-machined ultrasonic transducers (MUTs) fabricated in MEMS have shown good consistency in miniaturized geometric structures. Thus, MUTs are suitable to realize high-frequency phased arrays for compact ultrasonic systems. Compared with a capacitive MUT (CMUT), a piezoelectric MUT (PMUT) has the advantages of no DC bias, a linear relationship between the voltage and displacement, and enhanced acoustic transmission efficiency with larger displacement, etc. [9,11,12]. Moreover, with the reduced radius and pitch of element, PMUT achieves a higher resonant frequency. In recent years, PMUT arrays in the megahertz range have been developed for ultrasonic fingerprint and intracardiac imaging applications. Parallel connected elements [13] and phased arrays [14] have been used to improve the transmission efficiency. In particular, AlN-based PMUTs are compatible with the CMOS process, providing a possibility to integrate CMOS circuitry with compact 2D PMUT arrays. Given the broad prospects for PMUT arrays, an accurate evaluation of their electromechanical‒acoustic performance is very important for the design and optimization of high-frequency PMUTs.

Many studies have been conducted on feasible models to predict the performance of PMUT, which operates in flexural modes with a piezoelectric thin film and a silicon passive layer. The conventional method is based on the plate theory of a uniform circular PMUT. Smyth et al. [15] adopted a Green’s function approach to solve the axisymmetric vibration modes of the circular plate and verified the modes with a PZT-based PMUT having a radius of 400 μm. Sammoura et al. [16] used a clamped boundary and a simply supported boundary to optimize the electrode size of circular bimorph plates and compared with the measurements of a 196.5-kHz PMUT. Dangi et al. [17] reported a system-level approach based on analytical lumped models for PMUTs below 1 MHz to include the effects of flexural rigidity and residual tension. From these publications, it is clear that plate theory is applicable to low-frequency PMUT elements with a large radius and pitch. 

In addition, some numerical simulations of PMUTs were proposed for structural design and physical analysis. Eriksson et al. [18] compared the modes of an 89-kHz flexural ultrasound transducer with the finite element method (FEM), plate theory, and measurements. Massimino et al. [19] used an axisymmetric 2D model based on FEM for a 100-kHz PMUT. Lu et al. [20] built a 2D axisymmetric model in simulating the electromechanical‒acoustic behavior of a PMUT without a substrate. In fact, 2D axisymmetric models for simplified cylindrical PMUT and 2D models for square PMUT reduced the degree of freedom dramatically, thereby saving calculation storage and time. Nevertheless, considering the resonant frequencies and mode shapes that are changed by various geometric combinations, 2D simulations cannot accurately analyze composite structures or arrays, especially considering the anisotropy of materials. Therefore, Massimino [19] used a 3D FEM model with two symmetric orthogonal axes to analyze the deflection and acoustic pressure of a 4 × 4 air-coupled array. Shieh et al. [21] adopted a hybrid boundary for a large array to simulate a 7 × 7 circular matrix array at a resonant frequency of 8.75 MHz without a continuous supporting frame. The boundary condition is simplified from three dimensions (x, y, z) to two dimensions (x, y), thus reducing the calculation time, and it can only adapt to a case where the piezoelectric layer and the passive layer are rectangular or circular. This method is not suitable for analyzing such a structure, namely, an array supported by a continuous frame with circular cavities. As the complex structure consumes more computing power and the computer becomes more efficient, the 3D FEM model is the most effective and intuitive method to analyze and optimize the PMUT and its array. Of the existing papers, few have discussed simulation models of high-frequency PMUT, and the models have always been simplified to all discs or cuboid stacks [18,20,21], while ignoring the effect of the substrate.

Compared to the previous literature, the models in this paper have the following advantages: (1) the geometrically combined 3D FEM model, which consists of a circular sandwiched piezoelectric structure, a passive vibration layer, and a thick substrate with a vacuum cavity; (2) the anisotropy of materials, where piezoelectric and elastic matrices are selected based on fabricated AlN PMUT arrays; (3) modelling high-frequency electrically connected PMUT arrays using periodic boundary conditions; (4) prediction of electromechanical‒acoustic performance loaded with air and water using multiphysics coupling. In this paper, the resonant frequencies and mode shapes are simulated with 3D models and verified using fabricated PMUT arrays. Compared to the analytical method, the 3D FEM models simulate the resonance mode shapes and electromechanical‒acoustic performance of high-frequency PMUTs more accurately. Furthermore, the 3D FEM model has good potential to analyze the transmission and reception performance of large-scale PMUT arrays.

This paper is organized as follows: Section 2 describes the PMUT array structure and 3D FEM models in terms of material parameters, boundary conditions, mesh partition, etc. The simulation method is also described. In Section 3, scanning electron microscope (SEM) images of fabricated PMUT arrays are presented, as well as the experimental setup. A comparison of FEM, the analytical method, and experiments are shown in Section 4. The measured resonant frequencies and mode shapes are compared to the simulation results. The electrical impedance of arrays with air and water loads are experimentally validated and the acoustic propagations of the connected PMUT array are predicted. Finally, conclusions on the use of the 3D FEM model for high-frequency PMUT are given in Section 5.

## 2. FEM Simulations

### 2.1. Structural Design

**Figure 1:**
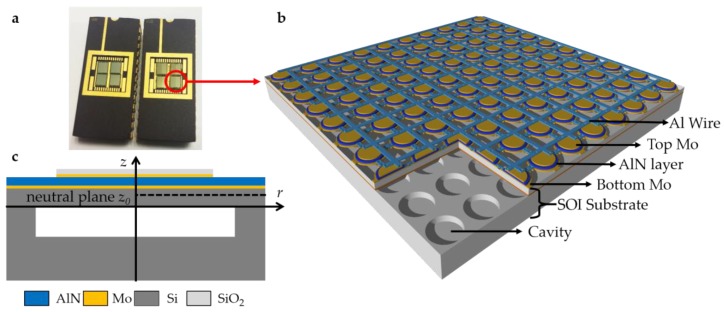
(**a**) Fabricated PMUT arrays in package; (**b**) 3D diagram of AlN PMUT array on cavity SOI; and (**c**) cross-sectional view of a PMUT for analytical method.

The PMUT array is implemented on a customized cavity SOI substrate. The packaged device and the 3D diagram are shown in Figure 1a and Figure 1b, respectively. Based on the flexural mode, the PMUT consists of a 1-μm AlN film, two 150-nm molybdenum (Mo) electrodes, and a 5-μm silicon membrane, where the AlN film is a piezoelectric active layer and the silicon membrane is a vibrational passive layer. As shown in Figure 1b, the Mo/AlN/Mo sandwiched structure is deposited on the membrane, which itself is a part of the SOI substrate, having a thickness of 715 μm. A cavity of 20 μm in depth is customized below the silicon membrane of the SOI substrate. The corresponding geometric parameters of the 16-MHz PMUT array are summarized in Table 1 for 3D FEM models and fabricated samples. 

When driven by the input voltage across the electrodes, the unbalanced moment induced by the piezoelectric effect causes the whole membrane structure to vibrate. The radii of stacking layers are determined with resonant frequency, material properties, and applied loads such as coupling layer and propagation medium. Normally, the pitch of the array elements needs to be between half and one wavelength (*λ*) of the ultrasound in the propagation medium, according to acoustic imaging applications [11]. In addition, the maximum vibration displacement of PMUT is obtained at the first resonant frequency (*f*_1_). The wavelength in the load medium (*λ = v_load_/f*_1_) is determined with the first resonant frequency and the ultrasound velocity (*v_load_*). For this reason, the pitch of the 16-MHz PMUT array is designated as 70 μm, which is 75% of the wavelength (*λ* = 95 μm) in water (*v_water_ ≈* 1500 m/s).

### 2.2. 3D FEM Model

**Figure 2:**
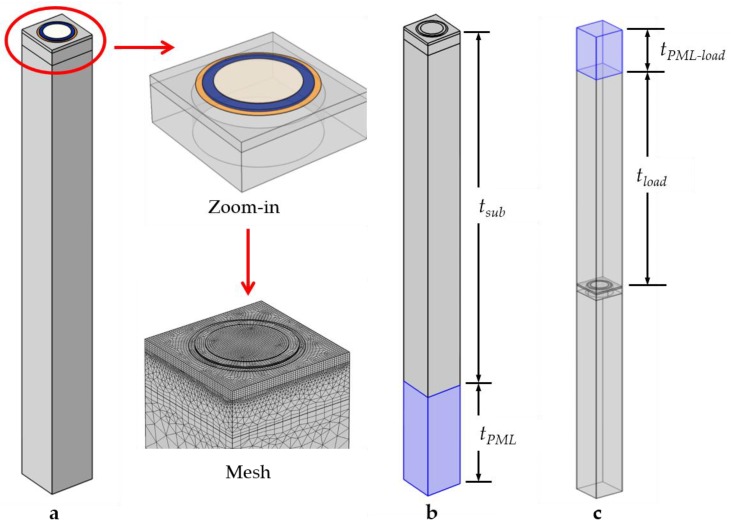
3D models with periodic boundary conditions using COMSOL v5.3a: (**a**) An element of the PMUT array, followed by a zoomed-in view and mesh partition of the detailed structure; (**b**) the element with a PML (in light blue) of substrate; (**c**) the element with an acoustic domain and an acoustic PML.

Three 3D FEM models are constructed using the abovementioned geometric parameters. These models take into account the sandwiched structure, the cavity under the passive layer, the substrate, and the passivation layer introduced by the process. In order to simulate the PMUT of connected array elements, periodic boundary conditions are applied. Figure 2a illustrates Model (a) in one period of the array, i.e., one PMUT element, for solid mechanical simulation in which the AlN layer and Mo electrodes are highlighted in blue and yellow, respectively. A zoomed-in view and mesh partition are shown. Based on Model (a), a perfectly matched layer (PML) of silicon in light blue is added to the bottom of the substrate in Figure 2b. Model (b) is used to distinguish the flexural modes of the silicon membrane, driven by the sandwiched structure, from the thickness modes of the substrate (*t_sub_* = 715 μm). The thickness (*t_PML_*) and typical wavelength (*λ_PML_*) of a PML are determined from the wavelength at first resonant frequency (*f*_1_ = 16 MHz), as *t_PML_* = 200 μm and *λ_PML_* = 500 μm according to *v_Si_* = 8433 m/s in silicon. In Figure 2c, Model (c) contains an acoustic domain (*t_load_*) and an acoustic PML to simulate the electrical impedance and acoustic field with air and water load (*t_PML-load_*), respectively. The PMUT array is assumed to operate at room temperature (*T_ref_* = 293.15 K) and 1 atm. Simulation under different loads is necessary in electromechanical‒acoustic analysis since the resonant frequency and energy conversion depend on the acoustic impedance of the load medium. In order to simulate the acoustic pressure and propagation of the connected PMUT array, acoustic periodic boundaries are also applied in the acoustic domain, where the thickness (*t_load_*) is set to multiple wavelengths of the load material, as *t_load_* = 750 μm. According to *v_air_* = 343 m/s and *v_water_* = 1500 m/s, the thickness (*t_PML-load_*) of acoustic PML is set to 50 μm in air and 150 μm in water, respectively, to simulate an infinite acoustic propagation medium without reflection. 

### 2.3. Simulation Method

The PMUT element is simulated in the following steps. The first is to predict the resonant frequencies and mode shapes of the PMUT element with Model (a) through an electromechanical analysis. The second is to simulate the excitation modes of Model (b) without substrate reflection. The third is to perform electromechanical‒acoustic simulations with Model (c) to predict the electrical impedance and acoustic field of PMUT with different loads.

These 3D numerical models contain important simulation parameters, like resonant frequencies, electrical impedance, and electromechanical coupling coefficients, which are functions of geometric parameters and material properties. In the piezoelectric layer, the electromechanical behavior is described with the piezoelectric constitutive Equations (1) and (2) [10]:

(1) $$T=c^{E}S-e^{T}E$$

(2) D=εE+eS

where *T*, *S*, *D*, and *E* represent the mechanical stress, strain vectors, electric displacement, and electric field vectors, respectively. The material parameters *c^E^*, *e*, *e^T^*, and *ε* are the elasticity matrix, piezoelectric crystal stress matrix, stress transposed matrix, and dielectric permittivity matrix, respectively. Unlike 2D models, 3D FEM models achieve higher accuracy and take into account the material anisotropy. As a piezoelectric material that is commercialized and compatible with integrated circuits, AlN is used in the proposed array, and its elastic and piezoelectric constants are illustrated in Table 2 [22]. Moreover, an orthotropic matrix of a standard [110] silicon is used in these 3D models [23]. The stacks contain a piezoelectric layer, a silicon passive layer, and other layers in the process (i.e., top and bottom electrodes and a passivation layer). All layers are considered as linear elastic materials and are summarized in Table 2 with the necessary properties.

For large arrays, 3D FEM simulations of hundreds of PMUT elements are too difficult to use to predict the electromechanical‒acoustic performance at resonant frequencies due to the heavy load of computational resources. In this paper, the simplified 3D model of one PMUT element using periodic boundary conditions is equivalent to the connected PMUT array. The periodic boundary is set at four lateral planes of one element, which is repeated to form a 2D PMUT array with a continuous silicon substrate. The bottom boundary of the substrate causes reflections in the compact array. The simulated resonant frequencies and mode shapes contain the thickness modes of the substrate and vibration modes of the silicon membrane actuated with the piezoelectric layer. In order to distinguish between these different modes, frequency analysis is performed with a free boundary and a bottom PML of the substrate, which leads to total reflection and total absorption, respectively. The boundary of the cavity is set as free since the customized SOI wafer is vacuumed during the process. The electrical boundary conditions are 1 volt at the top electrode and ground at the bottom electrode. The electroacoustic performance of the PMUT is simulated using an acoustic‒structure boundary between a solid actuated structure and an acoustic domain. In this interaction boundary, momentum balance and energy balance are considered. The acoustic PML is applied to simulate an infinite acoustic domain so that the electrical impedance and acoustic distribution are determined without bottom reflection.

For FEM simulations, the mesh elements in each domain are determined with the resonant frequency and material properties, as shown in Figure 2 (mesh). The maximum size of the mesh elements is specified as one-fifth of the wavelength in a given material in order to resolve stress waves within the solid domain accurately. Different from a 3D model with tetrahedral meshes, for a thin plate, a minimum of three solid elements through the thickness direction is needed. Therefore, the proposed 3D model distributes five mesh layers in the *z* direction of the 1-μm piezoelectric layer and the 5-μm passive layer, respectively. The quadrilateral mesh elements are drawn over the *x‒y* plane and swept along the *z* direction, where the distribution number is 5. In order to ensure the stress continuity and accuracy of the FEM model, stretching boundary layers are applied between the material interfaces. Compared to layers of several microns, growing triangular meshes are used for the 715-μm substrate to reduce computation time. The meshes are encrypted in the continuous frame, where the stress is concentrated. For the PML in the solid mechanical domain and acoustic domain, the meshes are distributed into at least five layers in one wavelength to absorb the stress and pressure waves, respectively. As a result, the number of degrees of freedom is about 0.8 million in electromechanical analysis and 1.5 million in electromechanical‒acoustic analysis.

Based on the proposed 3D models, these numerical simulations are performed using COMSOL (Stockholm, Sweden) Multiphysics Software (COMSOL 5.3a). The mentioned electromechanical‒ acoustic simulation for one frequency value can be completed in 40 min using a computer equipped with an Intel (Santa Clara, CA, USA) Xeon CPU (E5-2640 V4) and 128G RAM.

## 3. Experiments

### 3.1. Fabricated PMUT Array

**Figure 3:**
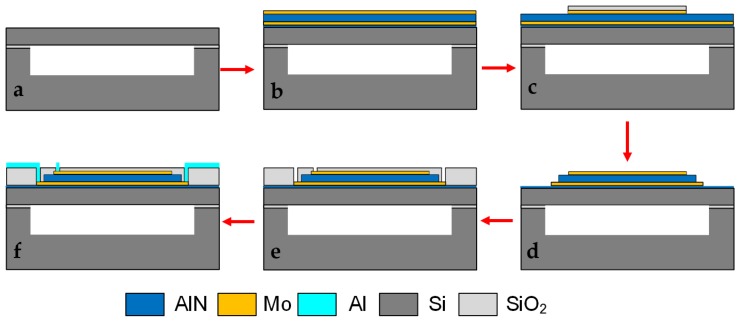
Micromachining process of the AlN-based PMUT array: (**a**) Customizing a cavity-SOI substrate; (**b**) depositing AlN seed layer/Mo/AlN/Mo layers; (**c**) using a SiO_2_ layer as a hard mask; (**d**) etching top electrodes, the AlN layer, and bottom electrodes by RIE in sequence; (**e**) depositing and etching a SiO_2_ insulation layer via holes; (**f**) depositing and patterning aluminum to form wires and bonding pads for a final array.

AlN PMUT arrays are fabricated on a double-side polishing SOI wafer with customized cavities [24,25]. In order to obtain high orientation and good roughness, a 20-nm AlN seed layer is first deposited on the SOI substrate. Then, the sandwiched Mo/AlN/Mo layers are deposited in different chambers using a Sigma (Allentown, PA, USA) FXP sputtering system. With a patterned silicon oxide (SiO_2_) layer as a hard mask for the subsequent etching step, the Mo/AlN/Mo microdisks are formed by reactive ion etching (RIE) with a combination of Cl_2_ and BCl_3_ gases and using Helium (He) to carry away the waste products. Thereafter, holes are opened in a 0.2-μm SiO_2_ insulation layer to connect the top and bottom electrode to the top metal layer. Finally, a 0.7-μm top metal (aluminum) layer is deposited and patterned to form wires and bonding pads. The micromachining process is illustrated in Figure 3.

**Figure 4:**
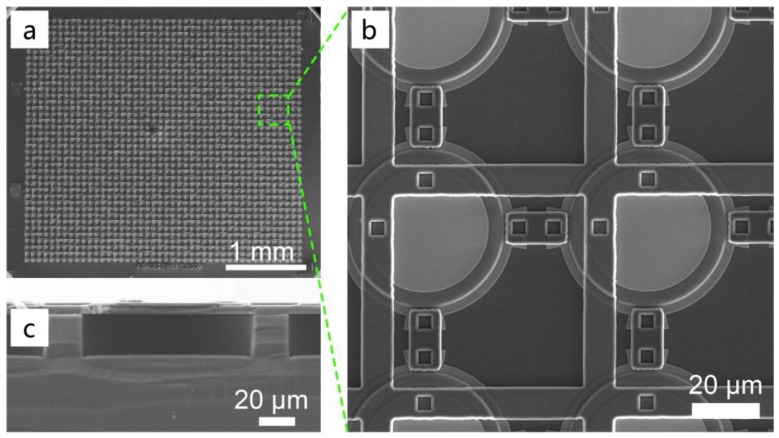
SEM images: (**a**) a top view of a 16 MHz AlN PMUT array (44 × 39), (**b**) a zoomed-in view of the PMUT array, and (**c**) a cross-sectional view of a PMUT element.

The 16-MHz PMUT array contains 44 × 39 elements in an area of approximately 3 mm × 2.7 mm, as shown in Figure 4a. Figure 4b shows a zoomed-in view of the electrically parallel array. Figure 3c shows a cross-sectional view of an element containing a sandwiched structure and a cavity.

### 3.2. Experimental Setup

**Figure 5:**
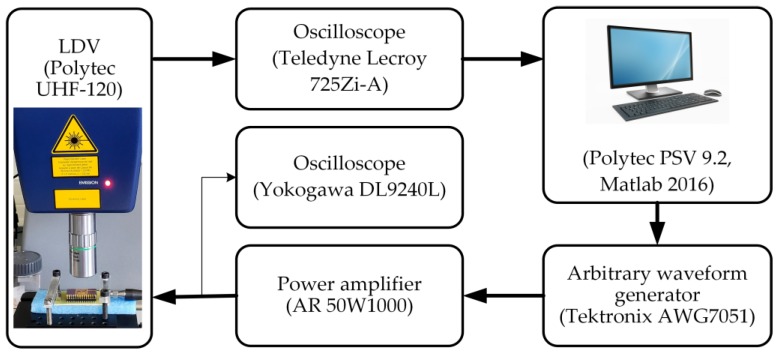
Functional diagram of the LDV setup for mode shape characterization of the PMUT array.

The resonant frequency and mode shape of the fabricated PMUT array are characterized using a laser Doppler vibrometer (LDV, UHF-120, Polytec GmbH, Waldbronn, Germany). This LDV is used to quantify vibration displacement point by point, and mode shapes are rebuilt by a mechanical scanning system. The measurement setup is shown in Figure 5. A transmission module delivers a wide-band linear chirp signal and a receiving module measures displacement in a synchronized manner with an optical heterodyne system. Input signals are generated using an arbitrary waveform generator (AWG7051, Tektronix, Beaverton, OR, USA) coupled with a power amplifier (50W1000, Amplifier Research, Tustin, CA, USA). The raw signal from the photodetector is collected with an oscilloscope (725Zi-A, Teledyne Lecroy, Chestnut Ridge, NY, USA) at a sampling rate of 40 GS/s. Furthermore, the in-phase quadrature (IQ) vibrational data demodulation and mode shape presentation are effectuated on a controlling personal computer (PC) using Polytec PSV 9.2 software. An additional oscilloscope (DL9240L, Yokogawa, Tokyo, Japan) is used to monitor the input signal as a reference for the vibrational output.

The electrical impedance loaded with air and water is measured using a vector network analyzer (ZVA 8, Rohde & Schwarz, Munich, Germany). The fabricated PMUT arrays, which can be seen in Figure 4, are packaged in a ceramic DIP using gold wire bonding (the same as is shown in Figure 1a).

## 4. Results and Discussion

### 4.1. Comparison of Analytical Method, FEM, and Experimental Results

In general, the resonant frequencies of PMUT are predicted by the well-known plate theory [11]. Based on clamped boundary condition, the first resonant frequency can be calculated by:

(3) $$f_{1}=\frac{{\left(3.19\right)}^{2}}{2\pi r^{2}}\sqrt{\frac{D}{I_{0}}}$$

where *r*, *D*, and *I_0_* are the radius, equivalent flexural rigidity, and surface density of a multilayer diaphragm, respectively (see Appendix A). The frequency is dependent on the material properties and geometric parameters, like neutral plane *z_0_* and radius *r* (in Figure 1c). Since thicknesses and material properties are unified using standard MEMS processes, the preferred resonant frequency for various applications is obtained by adjusting the diaphragm radius. In this paper, four PMUT arrays with different radii are fabricated on the same wafer using AlN micromachined processes. Corresponding FEM models are also built for comparisons. These simulation results are compared with experimental results in Table 3, where the measured first resonant frequency is the average of five samples.

**Figure 6:**
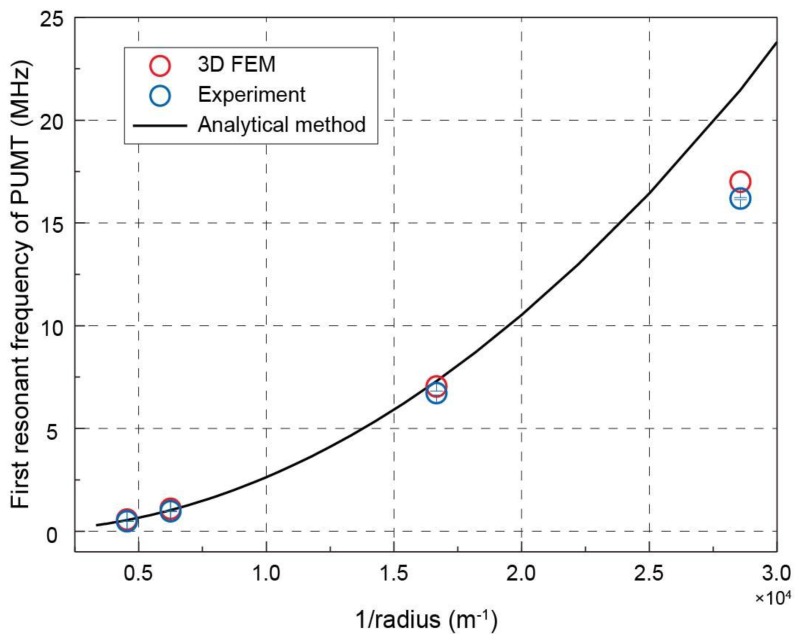
Comparison of the first resonant frequency of PMUT with different radii: obtained from 3D FEM simulations, experiments and analytical method. The error bar for each blue point represents standard deviation from five samples.

Figure 6 summarizes the first resonant frequency of PMUT with different radii obtained from the analytical method, 3D FEM model, and experiments (listed in Table 2). The calculation is performed according to Equation (3) using the material properties listed in Table 1. For low-frequency PMUTs, the results of the analytical method show good consistency with the measured resonant frequencies. However, for high-frequency PMUTs, the difference between analytical and experimental results increases from 2.8% to 25%. As the frequency increases, the radius and pitch of the array quickly converge. In this case, only the 3D FEM model predicts the performance of PMUT well, and the resonant frequency difference can be controlled within 6%.

### 4.2. FEM Analysis of a High-Frequency PMUT Array

When the top and bottom electrodes of PMUT are charged, the piezoelectric layer is excited and the whole membrane structure vibrates as a resonator. To analyze the resonant peaks, frequency spectrums are simulated using Model (a) with a free boundary and Model (b) with PML under the substrate. These two models are simulated in the electromechanical domain without a load. Considering the computation time of a 3D model with millions of degrees of freedom, a frequency step of 100 kHz is chosen for simulated frequency spectrums. 

**Figure 7:**
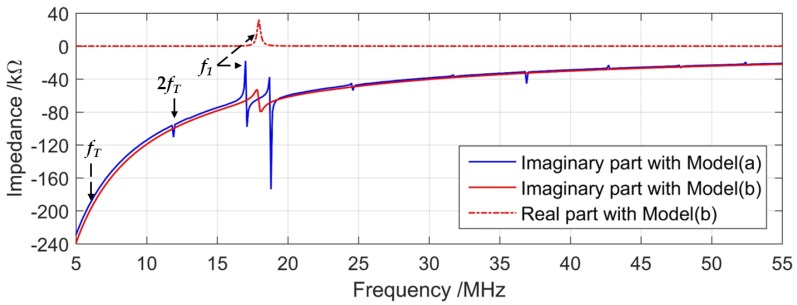
Electrical impedance of PMUT obtained from 3D FEM simulation with free boundary where the real part is null (**blue**) and PML (**red**) under substrate.

The electrical impedance spectrum of Model (a) shows a series of resonance modes, including membrane flexural modes and substrate thickness modes, as shown in Figure 7 with a blue curve. The frequency *f*_1_ corresponds to the fundamental resonance of the vibrating structure of the PMUT. The thickness modes of the silicon substrate (*t_sub_*) result in harmonics with a fundamental frequency of 5.9 MHz (*f_T_* = *v_Si_/*(2*t_sub_*)) in the spectrum, while these harmonics do not affect the effective working resonances of low-frequency PMUTs. In the blue curve of Figure 7, no significant peak appears at the frequency *f_T_*, since the mechanical deformation is small and the computation resolution, i.e., the frequency step, is not fine enough. Without energy loss, Model (a) only includes the imaginary part of the impedance. 

The electrical impedance of Model (b) consists of the real and imaginary parts, as shown in Figure 7 with red curves. As the PML in Model (b) absorbs substrate reflections, there is no resonant peak of the thickness mode in the red curves. Only one obvious peak is shown, indicating the vibration behavior of the circular membrane. Since the PML absorbs part of the electrical energy, there is a corresponding peak in the real part. 

The mechanical resonances take place at the corresponding frequency of zero impedance. For the blue curve corresponding to the free boundary condition, the mechanical resonance occurs at the electrical impedance null (zero), while with PML, the energy transduction maxima, i.e., resonance, occurs at peaks of near zero impedance. The first resonant frequency (*f*_1_) shifts by 1 MHz compared to that of Model (a). As a resonator, the resonance and antiresonance of the PMUT are very close and they can be distinguished at 17.0 MHz and 17.01 MHz for the fundamental resonance of the membrane structure with a finer frequency step of 10 kHz.

**Figure 8:**
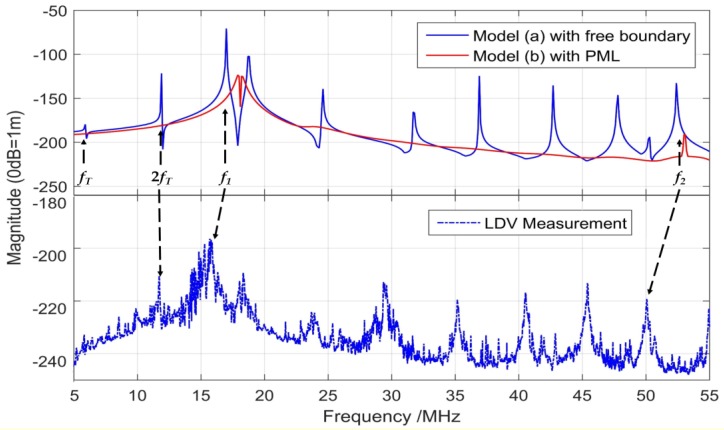
Displacement of PMUT obtained from 3D FEM simulations (above) and LDV measurements (below). In simulation results, Model (**a**) with free boundary is in blue and Model (**b**) with PML is in red.

In order to validate the mechanical resonances, the vibration displacement is determined at the surface center of these 3D models and compared to the results of the LDV measurements on the sample surface, as shown in Figure 8. The mechanical resonance with the maximum displacement takes place at frequency *f*_1_ and other modes appear in the frequency range of 5 to 55 MHz.

Consistent with the resonances of simulated electrical impedance in Figure 7, the solid blue curve in Figure 8 with free boundary condition illustrates the vibration displacement of resonances including membrane flexural modes and substrate thickness modes. The solid red curve shows the simulated displacement of Model (b), with PML absorbing the substrate reflections; therefore, only two displacement peaks appear in the spectrum, indicating the possible membrane flexural modes named *f*_1_ and *f*_2_. Model (b) is useful to demonstrate the membrane flexural mode, while Model (a) is helpful for indicating the overall phenomenon of the array. The comparison between Model (a) and Model (b) leads to better guidance on the geometric design and optimization of desired high-frequency PMUTs.

The experimental frequency spectrum of displacement is shown in the lower half of Figure 8, which is measured using the LDV setup (in Figure 5). The resonance mode shapes of one PMUT are recorded and rebuilt using Polytec PSV 9.2 software. The dotted blue curve in Figure 8 includes consistent resonant peaks compared with the solid blue curve, in which the maximum peak (*f*_1_) represents the first flexural mode of membrane. Corresponding to the thickness modes of the substrate, other peaks are repeated at an interval of 5.9 MHz in both curves. The measured curve (dotted blue line) shifts to the left slightly compared to the simulated one (solid blue line), i.e., the measured resonant frequencies are slightly smaller than the simulated ones. The amplitude of the remaining measured modes is 10~15 dB less than that of the frequency *f*_1_. Compared to the simulated displacement spectrum, the measured curve includes many burrs and a noise floor of ‒250 dB due to environmental and experimental noise. The numerical and experimental resonance mode shapes are summarized in Table 4. The figures in the second row represent the mode shapes simulated by the 3D FEM model with periodic boundaries. The figures with rainbow color measured by LDV setup are highly consistent with 3D FEM model results. 

It needs to be emphasized that the resonant frequencies and mode shapes from the 3D FEM model are in better agreement with the experimental results compared to those from the analytical method, although some differences still exist in the 3D FEM simulation. These differences could be due to the following factors: (1) the weight of the vibration structure increases due to electrode contact pads and wiring; (2) the material parameters used in FEM are slightly distinguished from the practical fabricated values; (3) the process errors are ignored compared to the geometric parameters of the designed PMUT.

### 4.3. Electromechanical‒Acoustic Analysis of a High-Frequency PMUT Array

The acoustic radiation from the PMUT into the loaded medium (air or water) reflects on the real part of electrical impedance. The electrical impedance of PMUT loaded with air and water is determined using Model (c). Since electrical boundary conditions are adopted for one PMUT, the simulated real part at the first resonance is about 10 kΩ with air load and 1.5 kΩ with water load. Based on the principle of circuit parallelism, the electrical impedance obtained from 3D FEM simulation predicts all 1716 PMUT elements connected in parallel, and the peak value of real part is about 6 Ω at 17.0 MHz with air load and 1 Ω at 14.1 MHz with water load. 

The electrical impedance characteristic of the PMUT array is verified with a vector network analyzer. Air and nonconductive deionized water (DI) are used in the measurements. When the PMUT array is immersed in water, the resonant frequency is reduced due to the water load acting as an additional mass. The measured electrical impedance of connected PMUT array is 15.9−j30 Ω at 15.8 MHz resonant frequency with air load and 4.72−j25 Ω at 13.9 MHz with water load. 

**Figure 9:**
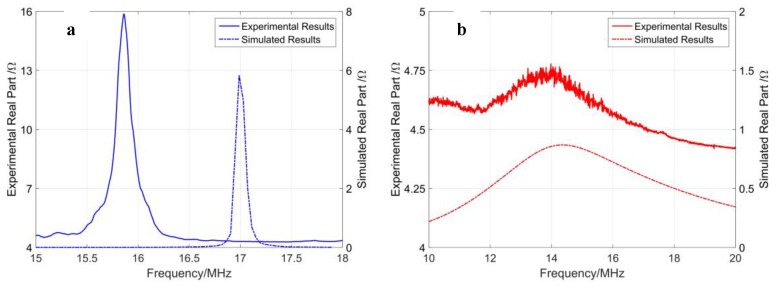
Electrical impedance comparison of the results using 3D FEM model and experimental results loaded with air (**a**) and water (**b**).

The simulated electrical impedance is compared with the corresponding measured ones, as shown in Figure 9, where blue curves indicate air load and red curves indicate water load. The electrical impedance obtained from 3D FEM simulation agrees with the measured results of the connected PMUT array. They share the same impedance order of magnitude at resonance, while the specific values vary depending on the samples. The first resonant frequency of FEM simulation shifted from 17 MHz with air load to 14.1 MHz with water load, while in experiments it changed from 15.8 MHz to 13.9 MHz. The measured impedance includes a floor of 4.2 Ω, which results from the resistance of the wires and pads.

**Figure 10:**
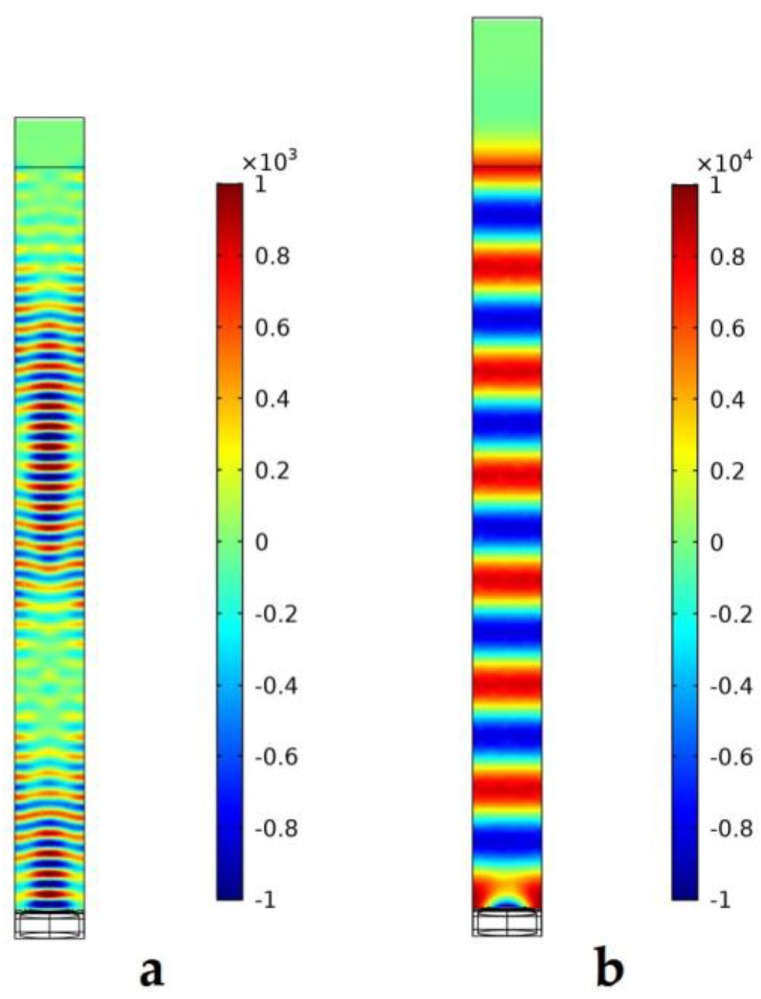
Simulated acoustic field in air (**a**) and in water (**b**) using Model (**c**).

In addition, 3D FEM models are used to predict the transmission sensitivity of the PMUT array with acoustic periodic boundary conditions. When charging 1 volt between the top and the bottom electrode, the simulated acoustic field using Model (c) in air and water is presented in Figure 10a,b, respectively. The simulated wave propagation of the PMUT in air and in water shows different wave numbers in the acoustic domain since the wavelength is about 20 μm in air and 100 μm in water at the corresponding resonant frequency. The acoustic impedance of air is much smaller than that of water, causing the PMUT to transmit more energy in water. As a result, the maximum acoustic pressure is about 1000 Pa in air at 17 MHz, and 10,000 Pa in water at 14.1 MHz. As shown in Figure 10b, the connected PMUT array emits uniform plane waves in water, which is suitable for ultrasonic plane wave application. In Figure 10a, interference occurs in air with the acoustic periodic boundary conditions, since the wavelength in air is smaller than that in water and the directivity is more selective. Moreover, the attenuation of the high-frequency ultrasonic wave in air is higher than that in water [26]. Therefore, high-frequency PMUT arrays are suitable for in vivo and high-resolution imaging with a load.

Three 3D FEM models determine the resonant frequencies and mode shapes and predict the electrical and acoustic performance of the connected PMUT array. Compared with conventional analytical methods, 3D FEM models demonstrate more electromechanical‒acoustic details, and are consistent with the sample experiments. Nevertheless, the main limitations of the proposed model include: (1) the time-consuming nature; (2) the enormous amount of computational resources required; and (3) the difficulties in the collaborative design of ultrasonic circuit system. These need to be continuously optimized and improved in subsequent simulation studies.

## 5. Conclusions

Three 3D FEM models with periodic boundary conditions are utilized to simulate high-frequency AlN-based PMUT arrays on cavity SOI. All the geometric parameters and material properties are chosen with reference to a fabricated 16-MHz PMUT array. The electromechanical simulation is performed to analyze the resonant frequencies and mode shapes of the PMUT. The electromechanical‒acoustic model is built to predict the electrical impedance and acoustic propagation of a PMUT array loaded with air and water. The FEM simulation results are verified by the experimental results. For high-frequency PMUTs, the frequency difference between FEM and the experimental results of the PMUT is controlled within 6%, which is just one-third of that between the analytical method and the measurement. The material anisotropy is taken into account, which gives rise to the higher accuracy of the 3D FEM results. For the design and optimization of high-frequency PMUT arrays, the 3D FEM model is suitable for simulating electromechanical‒acoustic performance and optimally adjusting the geometric parameters to meet different application requirements. Further experiments (e.g., ultrasound intensity measurements) are being performed to test the directivity and pressure of the PMUT array and verify the simulation results. These 3D models can also be employed to analyze the transmission and reception performance of PMUT arrays for future compact ultrasonic systems.

## Figures and Tables

**Table 1:** Geometric parameters for 3D FEM modeling and fabrication of 16-MHz PMUT arrays.

Material	Top Mo Electrode	AlN	Bottom Mo Electrode	Silicon Membrane	Cavity	Substrate
Radius (μm)	20	25	29	-	30	-
Thickness (μm)	0.15	1	0.15	5	20	715

**Table 2:** Material properties of AlN, Si, Mo, and SiO_2_ in 3D FEM models.

Property	Symbol	AlN	Si	Mo	SiO_2_
Density (kg/m^3^)	*ρ*	3512	2329	10,200	2200
Poisson ratio	*ν*	0.3	0.064	0.3	0.17
Young’s modulus (GPa)	*Y*	330	170	312	70
Elastic stiffness matrix (GPa) $\left[\begin{array}{cccccc} c_{11} & c_{12} & c_{13} & & & \\ c_{12} & c_{11} & c_{13} & & & \\ c_{13} & c_{13} & c_{33} & & & \\ & & & c_{44} & & \\ & & & & c_{44} & \\ & & & & & c_{66} \end{array}\right]$	*c*_11_	345	195		
	*c*_12_	125	36		
	*c*_13_	120	64		
	*c*_33_	395	166		
	*c*_44_	118	80		
	*c*_66_	110	51		
Piezoelectric stress matrix (C/m^2^) $\left[\begin{array}{ccccc} & & & & e_{15}\\ & & & e_{15} & \\ e_{31} & e_{31} & e_{33} & & \end{array}\right]$	*e*_31_	−0.58			
	*e*_33_	1.55			
	*e*_15_	−0.48			
Dielectric permittivity (-)	*ε/ε*_0_	11			

**Table 3:** First resonant frequency of PMUT arrays with 3D FEM model and experiments

Radius (μm)	First Resonant Frequency (in Hz)		Difference
	3D FEM Model	Experiment	
220	536 k	486 ± 2 k	10%
160	1060 k	976 ± 4 k	8.6%
60	7.0 M	6.6 ± 0.1 M	6%
35	17.0 M	16.0 ± 0.2 M	6%

**Table 4:** Summary of the numerical and experimental resonance mode shapes.

Resonance	*f*_1_	*f*_2_
**Numerical**	17.0 MHz	52.4 MHz
	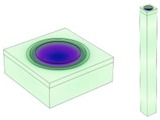	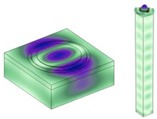
**Experimental**	15.8 MHz	50.1 MHz
	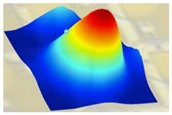	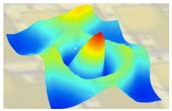

## Appendix A. Principle of Operation of Plates

The motion equation of the PMUT vibration is dependent on the piezoelectric moment *M_P_*, the pressure amplitude *P_w_*, and the displacement *w* in the *z* axis, shown in [27]:

(A1) $$\left(\nabla^{4}-\gamma^{4}\right)w=\frac{1}{D}\left(\nabla^{2}M_{P}+P_{w}\right)$$

where *γ* is a constant determined by the mode shape of the resonant frequency.

(A2) $$r^{4}=\frac{\omega^{2}I_{0}}{D}$$

(A3) $$D=\sum_{i=1}^{n}\frac{Y_{i}}{3\left(1-\nu_{i}^{2}\right)}\left[{\left(z_{i}^{2}-z_{0}^{2}\right)}^{3}-{\left(z_{i-1}^{2}-z_{0}^{2}\right)}^{3}\right]$$

where *z_i_* is the total thickness of the *i*th layer from plane *z* = 0, *a* is the radius of the diaphragm, and *Y_i_* and *v_i_* are the Young’s modulus and Poisson’s ratio from the *i*th layer material, respectively. The neutral plane *z*_0_ is conventionally defined as:

(A4) $$z_{0}=\frac{1}{2}\frac{\sum_{i=1}^{n}\frac{Y_{i}\left(z_{i}^{2}-z_{i-1}^{2}\right)}{1-\nu_{i}^{2}}}{\sum_{i=1}^{n}\frac{Y_{i}h_{i}}{1-\nu_{i}^{2}}}$$

The surface density *I*_0_ is the sum of the surface densities of each film, shown as follows:

(A5) $$I_{0}=\overset{n}{\underset{i=1}{\sum }}p_{i}h_{i}$$

where *ρ_i_* and *h_i_* represent the volume density and the thickness of the *i*th layer, respectively.
